# Development of an optimal imaging strategy for selection of patients for affibody-based PNA-mediated radionuclide therapy

**DOI:** 10.1038/s41598-018-27886-0

**Published:** 2018-06-25

**Authors:** Anzhelika Vorobyeva, Kristina Westerlund, Bogdan Mitran, Mohamed Altai, Sara Rinne, Jens Sörensen, Anna Orlova, Vladimir Tolmachev, Amelie Eriksson Karlström

**Affiliations:** 1 Department of Immunology, Genetics and Pathology, Uppsala University, Uppsala, Sweden; 2 Department of Protein Science, School of Engineering Sciences in Chemistry, Biotechnology and Health, KTH Royal Institute of Technology, Stockholm, Sweden; 3 Department of Medicinal Chemistry, Uppsala University, Uppsala, Sweden; 4 Nuclear Medicine and PET, Department of Surgical Sciences, Uppsala University, Uppsala, Sweden; 5 Medical Imaging Centre, Uppsala University Hospital, Uppsala, Sweden; 6 Science for Life Laboratory, Uppsala University, Uppsala, Sweden

## Abstract

Affibody molecules are engineered scaffold proteins, which demonstrated excellent binding to selected tumor-associated molecular abnormalities *in vivo* and highly sensitive and specific radionuclide imaging of Her2-expressing tumors in clinics. Recently, we have shown that peptide nucleic acid (PNA)-mediated affibody-based pretargeted radionuclide therapy using beta-emitting radionuclide ^177^Lu extended significantly survival of mice bearing human Her2-expressing tumor xenografts. In this study, we evaluated two approaches to use positron emission tomography (PET) for stratification of patients for affibody-based pretargeting therapy. The primary targeting probe Z_HER2:342_-SR-HP1 and the secondary probe HP2 (both conjugated with DOTA chelator) were labeled with the positron-emitting radionuclide ^68^Ga. Biodistribution of both probes was measured in BALB/C nu/nu mice bearing either SKOV-3 xenografts with high Her2 expression or DU-145 xenografts with low Her2 expression. ^68^Ga-HP2 was evaluated in the pretargeting setting. Tumor uptake of both probes was compared with the uptake of pretargeted ^177^Lu-HP2. The uptake of both ^68^Ga-Z_HER2:342_-SR-HP1 and ^68^Ga-HP2 depended on Her2-expression level providing clear discrimination of between tumors with high and low Her2 expression. Tumor uptake of ^68^Ga-HP2 correlated better with the uptake of ^177^Lu-HP2 than the uptake of ^68^Ga-Z_HER2:342_-SR-HP1. The use of ^68^Ga-HP2 as a theranostics counterpart would be preferable approach for clinical translation.

## Introduction

Standard treatment options for primary cancer include surgery and external radiation therapy, often in combination with (neo)adjuvant chemotherapy. However, patients with distant metastases at later stages of cancer need more advanced and selective systemic treatment approaches, such as targeted therapy.

Human epidermal growth factor 2 (Her2) is a transmembrane tyrosine kinase receptor which is overexpressed in about 20% of breast cancer and in 4–53% (average 18%) of gastric and gastroesophageal cancer cases^1,2^. Several Her2-targeted treatments have been shown to be effective in patients with Her2-positive breast and gastroesophageal cancer and provided significantly improved patient survival^3–6^. Her2-targeted immunotherapy using monoclonal antibody (mAb) trastuzumab together with chemotherapy has become a standard line of treatment for patients with Her2-positive breast cancer^7^. However, resistance is often developed during the first year of trastuzumab treatment^8^. In some cases, non-responders to trastuzumab treatment still have high Her2 expression.

Conjugation of mAbs to drugs, toxins or radionuclides is a way to potentiate their efficacy for targeted therapy of cancer. An advantage of radionuclides compared to drugs or toxins is the “crossfire” irradiation of cell clusters, which allows to overcome intratumoral target expression heterogeneity and necessity to deliver a cytotoxic agent to every single malignant cell^9^. Additionally, radionuclides can be used for treatment of multidrug-resistant tumors. Radiolabeled mAbs provided clinical benefits for patients with lymphoma, but did not improve the survival of patients with solid tumors^10^. The large size of mAbs leads to their long residence time in circulation, slow extravasation and slow accumulation in the tumor, which increases exposure of healthy tissues to radiation and limits the dose delivered to the tumor. Decreasing the size of the targeting protein generally decreases healthy tissue uptake, improves its tumor penetration and increases the tumor-to-normal tissue ratios^11^. One approach to combine the excellent specificity and affinity of antibodies with the rapid clearance and good tissue penetration of small targeting agents is to use pretargeting^12^. In pretargeting, the delivery of radionuclide to the tumor is achieved in two steps. In the first step, the primary agent targeting a tumor-associated antigen is administered. After accumulation in tumor and clearance from blood, a secondary agent, binding with high affinity to the primary agent and carrying a radionuclide is administered.

The pretargeting approach is mainly applied for antibody-mediated targeting. We have proposed to apply it for the affibody-mediated targeting^13–15^. Affibody molecules are small (58 amino acids, 7 kDa) scaffold proteins engineered using one of the staphylococcal protein A domains. High-affinity binders against several cancer-associated targets, such as EGFR, Her2 and Her3 have been selected and successfully applied for molecular imaging of tumor xenografts *in vivo*^16^. In clinical studies, the anti-Her2 affibody molecule ABY-025 labeled with ^68^Ga provided specific high-contrast imaging and allowed whole-body quantification of Her2 expression in metastatic breast cancer^17^.

Short residence time in circulation and optimal tumor targeting make affibody molecules potentially suitable for use in radionuclide therapy. However, when affibody molecules undergo renal clearance they are reabsorbed and rapidly internalized in proximal tubuli of kidneys. For radiometal-labeled affibody molecules, accumulation of radioactivity in kidneys is several-fold higher than in tumors^18^. To overcome high kidney accumulation of radiolabeled affibody molecules, pretargeting approaches were evaluated^13–15,19^.

In one approach, the interaction between the primary and the secondary agent is mediated by peptide-nucleic acid (PNA) hybridization. Being an analogue of DNA with a pseudopeptide backbone, a PNA strand is capable of selective and rapid hybridization with a complementary PNA strand. In addition, PNAs are not immunogenic, not toxic, and stable to nuclease and protease degradation, meeting all requirements for a biocompatible system^20^.

**Figure 1:**
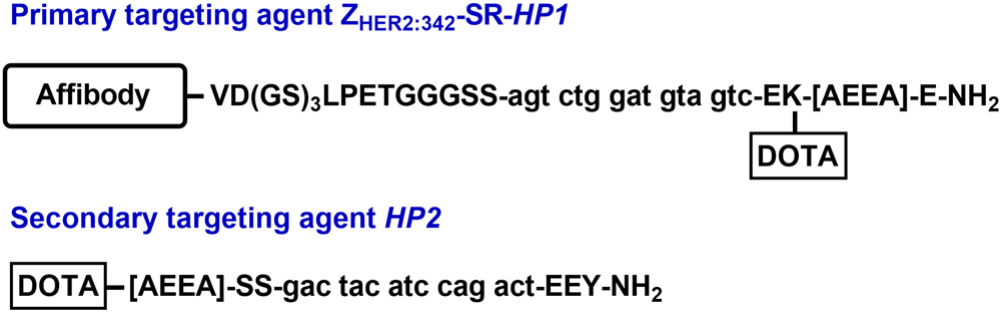
Affibody-based PNA-mediated pretargeting system consists of the primary (Z_HER2:342_-SR-HP1) and secondary (HP2) targeting agents.

The primary targeting agent used in this study consists of an anti-Her2 affibody molecule Z_HER2:342_ conjugated to a 15-mer PNA strand (Hybridization Probe 1, HP1) resulting in Z_HER2:342_-SR-HP1 (Fig. 1). The secondary agent is a complementary 15-mer PNA strand (Hybridization Probe 2, HP2). Both molecules have a DOTA chelator for labeling with radiometals. The primary agent Z_HER2:342_-SR-HP1 binds specifically and with picomolar affinity to Her2^14^. After binding, the internalization of the primary agent in Her2-expressing cells is slow (ca. 20% over 24 h), which makes it readily available for the secondary agent. The interaction between Z_HER2:342_-SR-HP1 and HP2 has a high association rate (1.7 × 10^5^ M^−1^ s^−1^) and slow dissociation rate resulting in a quick and stable binding of the secondary probe to the primary probe.

We have previously demonstrated the feasibility of affibody molecule-based PNA-mediated pretargeting *in vivo*^12,14^. Pre-injection of the primary probe Z_HER2:342_-SR-HP1 enabled specific tumor uptake of the secondary probe HP2 radiolabeled with ^125^I, ^111^In and ^177^Lu. In the case of therapeutic beta emitter ^177^Lu, a tumor-to-kidney ratio of 5 was achieved. This has prompted us to apply this pretargeting system for the radionuclide therapy of Her2-expressing tumors^19^. Six cycles of treatment with ^177^Lu-HP2 have resulted in significantly delayed tumor growth and improved median survival of mice with SKOV3 xenografts (66 days compared to 37 days in PBS-treated control). Importantly, this treatment protocol did not cause bone marrow or renal toxicity.

Given the successful outcome of the therapeutic study, this pretargeting approach is highly promising for theranostic applications in clinic. Labeling of the primary or secondary agents with the generator-produced positron-emitting radionuclide ^68^Ga would allow to visualize accumulation of these probes in tumors. The use of whole-body PET would enable to quantitatively assess the tumor accumulation and to address potential heterogeneity of the target expression. This should permit the selection of those patients who could benefit from Her2-targeted therapy and avoid overtreatment of patients with low pretargeting efficiency.

The goal of this study was to compare two imaging approaches, when either the primary targeting agent Z_HER2:342_-SR-HP1 or the secondary targeting agent HP2 was labeled with ^68^Ga, and to evaluate their efficiency for discrimination between high and low Her2 expression in a corresponding mouse model.

## Results

### Production, purification and labeling of PNA-based probes

A detailed description of production, synthesis and purification of the primary and secondary probes is reported in Westerlund *et al*.^13^. The primary targeting agent Z_HER2:342_-SR-HP1 bearing a DOTA chelator was labeled with ^68^Ga. Labeling was performed in 1.25 M sodium acetate buffer (pH 3.6) at 95 °C for 15 min with the radiochemical yield of 94 ± 1%. After incubation with 1000-fold molar excess of EDTA the radiochemical yield reduced to 85 ± 2%. The compound was purified using NAP-5 size-exclusion column resulting in 99.6 ± 0.4% radiochemical purity and 71 ± 4% isolated yield. Maximum specific activity of 2.1 MBq/µg (27.7 GBq/µmol, at the end of purification) was obtained.

The secondary targeting agent HP2 bearing a DOTA chelator was labeled with ^68^Ga. Labeling was performed in 1.25 M sodium acetate buffer (pH 3.6) at 95 °C for 15 min with the radiochemical yield of 94 ± 1%. After incubation with 1200-fold molar excess of EDTA a radiochemical yield of 90 ± 1% was obtained. Purification using NAP-5 size-exclusion column provided the compound with 98.6 ± 0.3% radiochemical purity and 53 ± 5% isolated yield. Maximum specific activity of 2.9 MBq/μg (15.0 GBq/µmol, at the end of purification) was obtained.

For biodistribution studies, HP2 was labeled with ^177^Lu with the radiochemical yield of 93.8 ± 0.4% and isolated yield of 56 ± 11% after NAP-5 size-exclusion purification. The radiochemical purity was 97 ± 1%.

### Binding and processing by Her2-expressing cells *in vitro*

**Figure 2:**
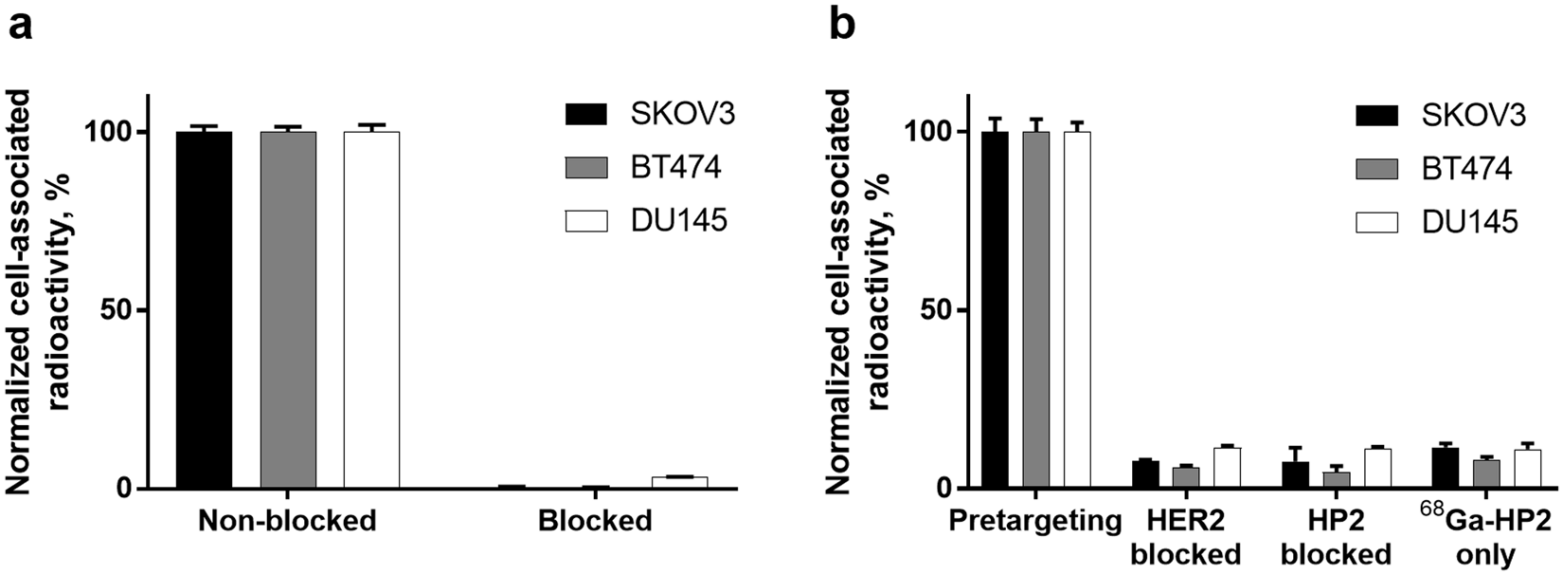
(**a**) *In vitro* binding specificity of the primary agent ^68^Ga-Z_HER2:342_-SR-HP1. In the control group, Her2 was blocked by adding 500-fold molar excess of non-labeled anti-Her2 Z_HER2:342_ affibody molecule. (**b**) *In vitro* binding specificity of the secondary agent ^68^Ga-HP2 pretargeting to Her2-expressing SKOV3, BT474 and DU145 cells. In the control groups, Her2 was blocked by adding 500-fold molar excess of non-labeled anti-Her2 Z_HER2:342_ affibody molecule, HP1 was blocked by adding 150-fold excess of non-labeled HP2. In the third control, no Z_HER2:342_-SR-HP1 was added. The data are presented as an average value from 3 samples ± SD.

Her2-binding specificity of the primary agent ^68^Ga-Z_HER2:342_-SR-HP1 was tested using a saturation assay in Her2-expressing SKOV3, BT474 and DU145 cells. The binding was significantly (p < 0.000001 for all cell lines) decreased when the cells were pre-incubated with the parental anti-Her2 affibody molecule (Fig. 2), demonstrating that the binding was specific and Her2-mediated. The level of cell-associated radioactivity in non-blocked SKOV3 and BT474 cells was higher than in DU145 cells, which is consistent with their Her2 expression level.

The specificity of ^68^Ga-HP2 *in vitro* binding to Z_HER2:342_-SR-HP1-pretreated Her2-expressing cells was confirmed in SKOV3, BT474 and DU145 cells (Fig. 2). The binding of ^68^Ga-HP2 was significantly (p < 0.00001 for all cell lines) decreased when Her2 receptors were saturated with parental anti-Her2 affibody molecule or when Z_HER2:342_-SR-HP1-treated cells were pre-incubated with a large excess of non-labeled HP2. Additionally, the binding of ^68^Ga-HP2 to cells without pre-incubation with the primary agent Z_HER2:342_-SR-HP1 was significantly (p < 0.00001 for all cell lines) decreased. This assay demonstrated that the pretargeting of the secondary agent was Her2-specific, PNA-mediated and depended on the pre-treatment with the primary agent.

**Figure 3:**
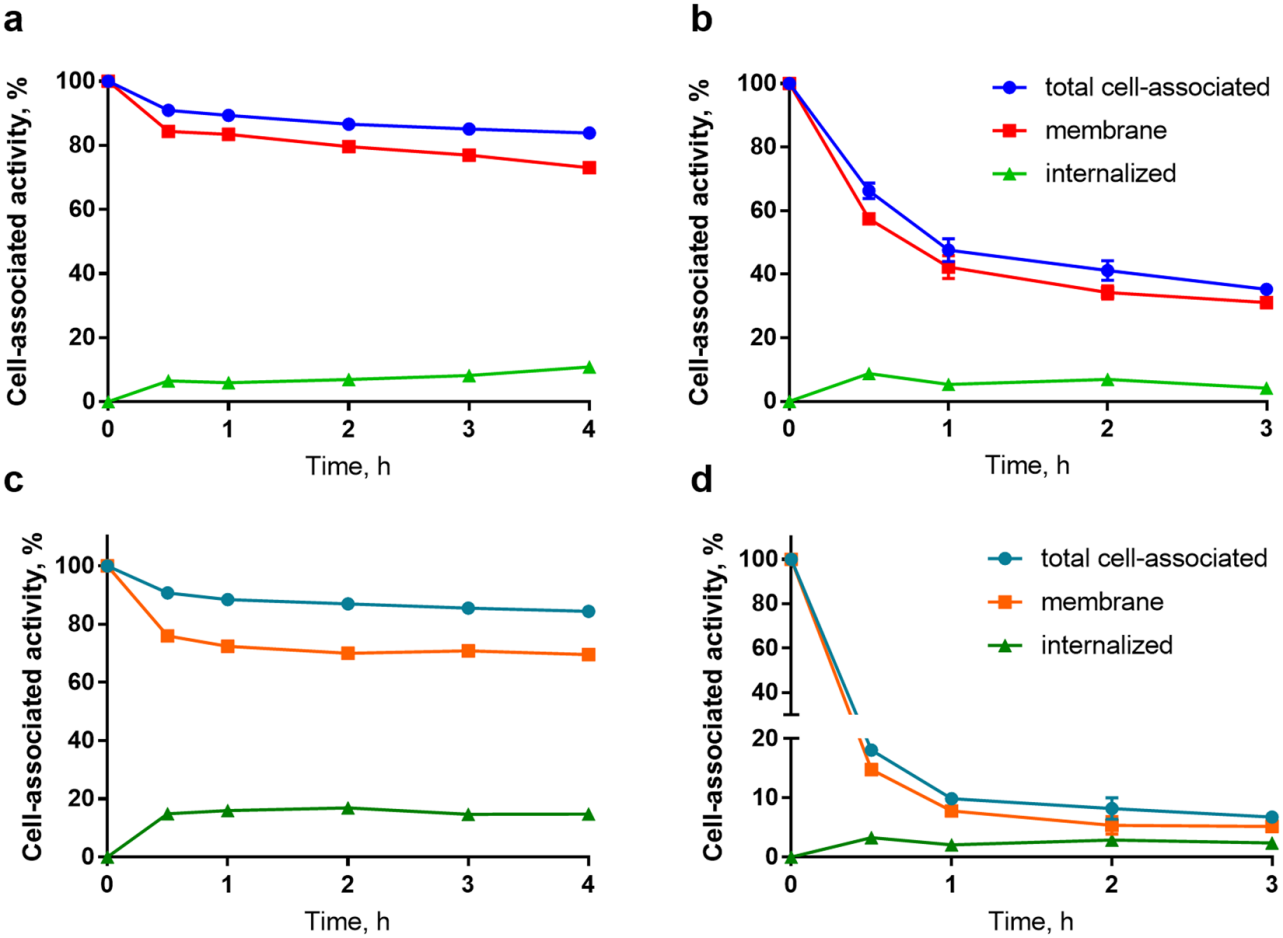
Cellular processing of ^68^Ga-Z_HER2:342_-SR-HP1 (**a**,**b**) and of Z_HER2:342_-SR-HP1:^68^Ga-HP2 complex (**c**,**d**) by Her2-expressing SKOV3 (**a**,**c**) and DU145 (**b**,**d**) cells. The data are presented as an average value from 3 samples ± SD.

Cellular processing of ^68^Ga-Z_HER2:342_-SR-HP1 by SKOV3 and DU145 cells after interrupted incubation is shown in Fig. 3. The retention of the radiolabeled primary agent on the cell membrane was higher in high-expressing SKOV3 cells than DU145 cells with low Her2 expression (77 ± 1% for SKOV3 vs. 31 ± 2% for DU145 at 3 h time point). The internalization of ^68^Ga-Z_HER2:342_-SR-HP1 was low in both cell lines. The internalized fraction in SKOV3 cells at 4 h was 11 ± 1% and in DU145 at 3 h was 4 ± 1%, which is in agreement with the previously reported values for the ^111^In-labeled analogue^14^ (in SKOV3 cells at 4 h it was 8.0 ± 0.3%).

Cellular processing of the Z_HER2:342_-SR-HP1:^68^Ga-HP2 complex had a similar pattern as the processing of the primary agent alone (Fig. 3). Initial release from the membrane after 1 h was followed by a plateau resulting in high retention of radioactivity by SKOV3 cells (ca. 80% of initially bound activity at 4 h). However, in DU145 cells with low Her2 expression the complex was rapidly released from the membrane with only 10% of total cell-associated activity at 1 h.

The binding strength of ^nat^Ga-Z_HER2:342_-SR-HP1 to Her2-expressing SKOV3 cells was compared to the binding strength of ^nat^In-Z_HER2:342_-SR-HP1 in a competitive binding assay using ^111^In-DOTA-Z_HER2:2395_^21^ as the displacement radioligand. The IC_50_ values for ^nat^Ga-Z_HER2:342_-SR-HP1 and ^nat^In-Z_HER2:342_-SR-HP1 were determined to be 24 ± 2 nM and 22 ± 1 nM, respectively (SI Fig. 1). No difference between the IC_50_ values was observed, which suggested that the affinity of gallium-labeled Z_HER2:342_-SR-HP1 is similar to the affinity of the indium-labeled conjugate.

### Animal studies

Initial assessment of ^68^Ga-HP2 biodistribution was performed in healthy NMRI mice. The biodistribution of ^68^Ga-HP2 and ^177^Lu-HP2 in NMRI mice at 1 and 2 h p.i. (SI Table 1) showed rapid and predominantly renal clearance of probes. The data for the ^177^Lu-HP2 biodistribution was in a good agreement with the previously reported values^12^. As it was earlier observed for ^111^In-HP2 and ^177^Lu-HP2, the radionuclide had a noticeable influence on biodistribution of the secondary probe. At both time points, ^68^Ga-HP2 had significantly higher blood retention than ^177^Lu-HP2 (0.21 ± 0.05 vs. 0.16 ± 0.03% ID/g at 1 h, p = 0.03), as well as higher uptake in liver, lung and kidneys.

To compare two approaches, direct targeting and pretargeting, the biodistribution of ^68^Ga-labeled primary and secondary agents was evaluated in BALB/C nu/nu mice bearing SKOV3 (high Her2 expression) and DU145 (low Her2 expression) xenografts (SI Fig. 3, Table 1). In both approaches, a high dose of the primary agent Z_HER2:342_-SR-HP1 (100 µg) was administered to saturate Her2 receptors and provide maximum discrimination between high and low Her2-expressing tumors^22^. The pretargeting protocol used in this study has been previously optimized for the affibody-based PNA-mediated therapy using ^177^Lu-HP2^19^. It was found that 16 h between the injections of primary and secondary agents and 3.5 µg of the secondary agent provided the highest tumor accumulation, low kidney uptake and optimal dose delivered to the tumor.

**Table 1:** Biodistribution comparison of ^68^Ga-Z_HER2:342_-SR-HP1 (100 µg), ^68^Ga-HP2 and ^177^Lu-HP2 in BALB/C nu/nu mice bearing SKOV3 (high Her2 expression) and DU145 (low Her2 expression) xenografts at 1 h p.i. Z_HER2:342_-SR-HP1 (100 µg) was injected 16 h prior to ^68^Ga-HP2 and ^177^Lu-HP2 (3.5 µg total) injections. The uptake is expressed as % ID/g and presented as an average value from 4 mice ± SD (5 mice ± SD for the pretargeting groups). Data for GI tract with content and carcass are presented as % of injected dose per whole sample. One-way ANOVA with Bonferroni’s multiple comparisons test was performed to find significant differences.

	^68^Ga-Z_HER2:342_-SR-HP1		Z_HER2:342_-SR-HP1 + ^68^Ga-HP2		Z_HER2:342_-SR-HP1 + ^177^Lu-HP2	
	SKOV3	DU145	SKOV3	DU145	SKOV3	DU145
blood	0.7 ± 0.1	0.6 ± 0.1	0.17 ± 0.04^*a*^	0.19 ± 0.03	0.19 ± 0.06	0.15 ± 0.03
lung	1.0 ± 0.2	0.8 ± 0.1	0.24 ± 0.04^*a*^	0.31 ± 0.06	0.29 ± 0.06	0.28 ± 0.06
liver	2.4 ± 0.4	2.1 ± 0.1	1.0 ± 0.1^*a,b*^	1.5 ± 0.1	0.73 ± 0.03 ^*d*^	1.09 ± 0.09
spleen	1.0 ± 0.3	1.0 ± 0.1	0.28 ± 0.03^*a*^	0.34 ± 0.08	0.14 ± 0.02	0.16 ± 0.04
kidney	315 ± 31	289 ± 35	9.5 ± 0.2^*a*^	12 ± 1	7.5 ± 0.4	6.7 ± 0.6
tumor	4.3 ± 0.9	1.3 ± 0.3	6.3 ± 1.5^*b,c*^	0.5 ± 0.2	12 ± 3^*d*^	0.5 ± 0.3
muscle	0.3 ± 0.1	0.24 ± 0.05	0.14 ± 0.08^*a*^	0.07 ± 0.02	0.09 ± 0.06	0.05 ± 0.03
bone	0.5 ± 0.1	0.4 ± 0.1	0.13 ± 0.03^*a*^	0.09 ± 0.01	0.05 ± 0.01	0.04 ± 0.03
GI tract	1.1 ± 0.3	0.8 ± 0.1	0.6 ± 0.2^*a*^	0.3 ± 0.1	0.4 ± 0.3	0.3 ± 0.1
carcass	7 ± 1	6 ± 1	3 ± 1^*a*^	3 ± 2	2 ± 1	3 ± 2
**Tumor-to-organ ratio**						
blood	6.2 ± 0.5	2.3 ± 0.3	40 ± 17^*a,b*^	3 ± 1	56 ± 18	4 ± 2
lung	4.5 ± 0.3	1.6 ± 0.3	27 ± 8^*a,b,c*^	2 ± 1	42 ± 15	2 ± 1
liver	1.8 ± 0.3	0.6 ± 0.1	7 ± 1^*a,b,c*^	0.4 ± 0.1	16 ± 4	0.5 ± 0.2
spleen	4 ± 1	1.3 ± 0.3	23 ± 5	1.7 ± 0.5	88 ± 27	3 ± 1
kidney	0.013 ± 0.001	0.004 ± 0.001	0.7 ± 0.2^*a,b,c*^	0.04 ± 0.01	1.5 ± 0.3	0.08 ± 0.04
muscle	16 ± 2	5 ± 1	71 ± 56	8 ± 2	176 ± 104^*d*^	10 ± 3
bone	9 ± 1	3.5 ± 0.4	52 ± 25^*c*^	6 ± 2	257 ± 101	18 ± 13

^*a*^Significant difference between ^68^Ga-Z_HER2:342_-SR-HP1 and ^68^Ga-HP2 uptake in SKOV3 group.

^*b*^Significant difference in ^68^Ga-HP2 uptake between SKOV3 and DU145 groups.

^*c*^Significant difference between ^68^Ga-HP2 and ^177^Lu-HP2 uptake in SKOV3 group.

^*d*^Significant difference in ^177^Lu-HP2 uptake between SKOV3 and DU145 groups.

In the direct targeting approach the biodistribution of ^68^Ga-labeled primary probe, ^68^Ga-Z_HER2:342_-SR-HP1, at 1 h p.i. was typical of affibody molecules, i.e. rapid clearance from blood and normal tissues and a high level of renal reabsorption. Tumor-associated radioactivity in the SKOV3 group was about three times higher compared to the DU145 group (4.3 ± 0.9 vs. 1.3 ± 0.3% ID/g, p = 0.0006).

In the pretargeting approach, injection of the unlabeled primary agent 16 h before the administration of ^68^Ga-HP2 resulted in approximately twelve-fold higher tumor uptake in the SKOV3 group compared to the DU145 group (6.3 ± 1.5 vs. 0.5 ± 0.2% ID/g) at 1 h p.i. Pretargeting provided significantly (p < 0.0001, one-way ANOVA test) higher tumor-to-organ ratios, i.e. six-fold higher tumor-to-blood, four-fold higher tumor-to-liver and fifty four-fold higher tumor-to-kidney ratios, compared to direct targeting in mice bearing SKOV3 xenografts.

To further evaluate if pretargeted imaging with ^68^Ga-HP2 could be used for the prediction of tumor uptake of therapeutic ^177^Lu-HP2, the biodistribution of ^177^Lu-HP2 was studied alongside with ^68^Ga-HP2 in the same mice (Table 1, SI Fig. 2). In SKOV3-bearing mice pretargeted with Z_HER2:342_-SR-HP1, tumor uptake of ^177^Lu-HP2 was about twice higher than ^68^Ga-HP2 (12 ± 3 vs. 6.3 ± 1.5% ID/g, p = 0.00006, one-way ANOVA test), while in DU145 tumors with low Her2 expression no difference in uptake was observed. No statistically significant differences (p > 0.05, one-way ANOVA test) between uptake of ^177^Lu-HP2 and ^68^Ga-HP2 was observed in healthy organs and tissues in SKOV3 group.

### Imaging

**Figure 4:**
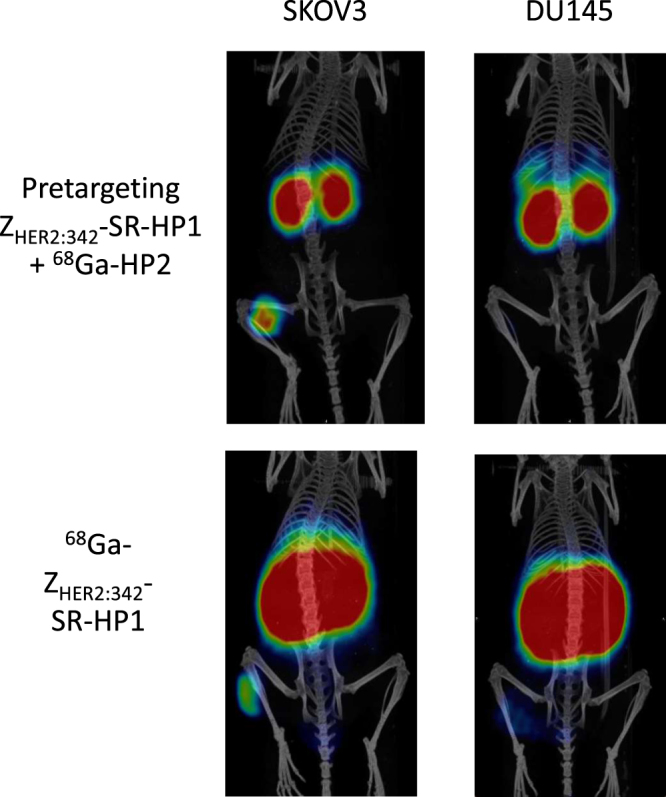
MicroPET/CT imaging of Her2-expressing SKOV3 (high Her2 expression) and DU145 (low Her2 expression) xenografts at 1 h after injection. Lower SUV threshold 0.07, upper SUV threshold 0.7.

PET/CT imaging confirmed the results of the biodistribution studies. Both direct targeting using ^68^Ga-Z_HER2:342_-SR-HP1 and pretargeting using Z_HER2:342_-SR-HP1 and ^68^Ga-HP2 were able to visualize Her2-expressing xenografts in mice (Fig. 4). In agreement with the *ex vivo* data, the uptake of radioactivity in SKOV3 xenografts was higher than in DU145 xenografts. In the direct targeting approach high accumulation of radioactivity was observed in kidneys, while the pretargeting provided decreased kidney uptake and increased tumor uptake of radioactivity. No noticeable uptake in other organs was detected.

## Discussion

Clinical PET/CT imaging using ^68^Ga-labeled ABY-025 demonstrated that this affibody molecule provides non-invasive whole-body quantification of Her2 expression in patients with metastatic breast cancer^17^. We have recently demonstrated that affibody-mediated pretargeted radionuclide therapy using ^177^Lu successfully delayed tumor growth and doubled median survival of mice bearing SKOV3 xenografts. Importantly, no acute toxicity or kidney damage was observed^19^. Promising results of pretargeted radionuclide therapy and clinical applicability of affibody molecules for imaging prompted us to develop a theranostic methodology for the affibody-based pretargeting treatment, where PET/CT imaging would guide the selection of patients for therapy.

The most straightforward way would be to base the decision concerning therapy on the results of quantitative imaging of Her2-expression using ^68^Ga-ABY-025. The assumption would be that the patients with high expression would have high tumor accumulation of the therapeutic probe. However, this approach does not take into account the differences in structures of ABY-025 and Z_HER2:342_-SR-HP1-DOTA leading to different capacity in penetrating the vasculature, potential differences in their accumulation in tumors and the differences in probe dosing between imaging and therapy. Another strategy would be the pretargeted theranostics using the positron-emitting radionuclide gallium-68. Such a strategy could be realized in two ways depending on the placement of the label. When ^68^Ga is placed on Z_HER2:342_-SR-HP1, PET/CT imaging would provide assessment of tumor accumulation of the primary agent and identification of patients with sufficiently high accumulation. Alternatively, the ^68^Ga label could be placed on the secondary agent. In that case, unlabeled primary agent is injected the day before the diagnostic secondary agent ^68^Ga-HP2. Patients with high tumor uptake of radioactivity would then proceed to pretargeted treatment. The first approach is simpler logistically, however, the second might better reflect the whole pretargeted delivery of a radionuclide to tumors.

In this study, we performed a side-by-side comparison of direct targeting using radiolabeled primary agent ^68^Ga-Z_HER2:342_-SR-HP1 and pretargeting using radiolabeled secondary agent ^68^Ga-HP2 for imaging of Her2 expression. To address a clinically relevant problem of discrimination between tumors with high and low Her2 expression we compared these two approaches in mice bearing SKOV3 (high Her2) and DU145 (low Her2) tumor xenografts.

The primary Z_HER2:342_-SR-HP1 and secondary HP2 probes carrying a DOTA chelator were labeled with ^68^Ga with good yields. Minor release of ^68^Ga under pre-purification EDTA challenge from both primary (ca. 10%) and secondary (ca. 5%) probes could be due to the presence of weak chelating sites that compete with DOTA for ^68^Ga. Despite this initial release of ^68^Ga, neither of the conjugates ^68^Ga-Z_HER2:342_-SR-HP1 and ^68^Ga-HP2 showed any signs of label loss *in vivo* (e. g. elevated bone uptake).

Gallium-labeled primary probe ^68^Ga-Z_HER2:342_-SR-HP1 retained binding specificity to Her2-expressing cells (Fig. 2a). Pretargeting specificity of the secondary probe ^68^Ga-HP2 was also confirmed (Fig. 2b). Ga-Z_HER2:342_-SR-HP1 had the same half maximal inhibitory concentration (IC_50_) (Supplementary Information (SI) Fig. 1) as In-Z_HER2:342_-SR-HP1 used in earlier studies^14^. An important factor for the success of pretargeting is the availability of the primary probe on the cell surface for the reaction with the secondary probe. The retention of the primary probe ^68^Ga-Z_HER2:342_-SR-HP1 on the surface of SKOV3 cells was high, which is in accordance with the previously reported data for ^111^In-Z_HER2:342_-SR-HP1^14^ and is typical for affibody molecules.

Labeling chemistry and the choice of radionuclide might have a profound effect on the biodistribution of peptides and small targeting molecules^12,23–26^. In our previous studies the primary agent Z_HER2:342_-SR-HP1-DOTA was labeled with ^111^In^14^.

Although both indium and gallium are trivalent metals, they have a different ionic radius and their complexes with DOTA have different coordination geometry. The complex of indium with the amide derivative of DOTA has a square-antiprismatic geometry with the amide oxygen involved in chelation, where indium is octacoordinated. The gallium-DOTA complex has a pseudo-octahedral geometry and gallium is hexacoordinated^27^. This leaves a free carboxyl group noninvolved in coordination and a different charge distribution compared to the complex with indium. Despite these differences, the uptake of the gallium-labeled primary agent in SKOV-3 xenografts (4.3 ± 0.9% ID/g) was similar to the indium-labeled analogue^14^ (5.9 ± 2.4% ID/g).

The secondary pretargeting agent HP2-DOTA was previously radiolabeled with ^111^In and ^177^Lu^12^. It was found that the radionuclide had a substantial effect on the biodistribution of this molecule. ^177^Lu-HP2 had a faster clearance from blood and normal organs, except kidneys, compared to ^111^In-HP2 already at 1 h p.i.

In this study, ^68^Ga-HP2 was excreted from blood slower than ^177^Lu-HP2 (Table 1 and SI Table 1) but faster than ^111^In-HP2 at 1 h p.i.^12^. In general, the uptake of ^68^Ga-HP2 in normal organs and tissues was higher than of ^177^Lu-HP2 at both 1 and 2 h p.i. (SI Table 1). High kidney uptake of ^68^Ga-HP2 (9 ± 2 vs. 4.6 ± 0.5% ID/g for ^177^Lu-HP2 at 1 h, p = 0.008) also suggested higher reabsorption rate of this molecule in kidneys compared to ^177^Lu-HP2. As discussed above, the difference in biodistribution could be attributed to the differences in ionic radii of the radionuclides and geometries of metal-DOTA complex. Lutetium has a larger ionic radius than gallium and the complex of Lu^3+^ and DOTA derivatives predominantly exists in a square antiprism geometry with different bond length compared to the indium-DOTA complex^21^. This phenomenon was also observed in preclinical studies of other theranostic pairs, e.g. ^68^Ga/^177^Lu-PSMA^28^ and ^67^Ga/^111^In/^177^Lu-NeoBOMB1^29^ in direct targeting or ^68^Ga/^111^In- IMP288 in different pretargeting systems^30,31^. Due to the differences in biodistribution in this mouse model, PET imaging with ^68^Ga-HP2 would not precisely predict the uptake of the therapeutic ^177^Lu-HP2, however, it should be further evaluated if these differences would translate to humans.

In this study, both direct use of ^68^Ga-Z_HER2:342_-SR-HP1 and pretargeted imaging using ^68^Ga-HP2 allowed discrimination between tumors with high and low Her2 expression (Table 1, Fig. 4). Direct targeting provided 3.3-fold difference between uptake in SKOV-3 and DU-145 xenografts, while pretargeting provided 11.8-fold difference. In clinical studies using ^68^Ga-labeled anti-Her2 affibody molecule, Standard Uptake Values (SUV) in lesions with confirmed Her2-status were approximately five times higher in Her2-positive metastases than in Her2-negative^17^. Notably, the clinical study indicated that among the metastases defined as Her2-positive by biopsies and immunohistochemistry the SUVs ranged from 6 to more than 40 at 2 h after tracer injection. In clinical practice, a single whole-body scan performed early after tracer injection would then be able to characterize each individual metastasis on a continuous scale regarding Her2 protein expression. Access to this directly obtainable information on clonal heterogeneity and tumor burden might lead to more optimal and earlier treatment decisions. The dramatically improved tumor-to kidney uptake ratio obtained with pretargeting in this study is promising. In the clinical study using direct imaging tumor-to-kidney ratios ranging from 1/10 to 1/1 were recorded, which appears to be much lower than in mice, suggesting that pretargeting could reduce kidney uptake even further in humans and indicates that kidney-sparing treatment using endogenous radiation with a theranostic approach might be one of the available therapeutic options.

Overall, imaging using ^68^Ga-HP2 provided better discrimination between tumors with high and low Her2 expression and better reflected the tumor uptake of the therapeutic counterpart, ^177^Lu-HP2. This method should be considered as the most promising for clinical translation.

## Methods

Buffers used for labeling were prepared from high-quality Milli-Q water and purified from metal contamination using Chelex 100 resin (Bio-Rad Laboratories, USA). ^111^InCl_3_ was purchased from Mallinckrodt Sweden AB (Stockholm, Sweden). Carrier-free ^177^LuCl_3_ was purchased from PerkinElmer (Waltham, MA, USA). Radioactivity was measured using an automated gamma-spectrometer with a NaI(TI) detector (1480 Wizard, Wallac, Finland). Her2-expressing SKOV3, BT474 and DU145 cells were purchased from the American Type Culture Collection (ATCC) and were cultured in complete RPMI-medium supplemented with 10% fetal bovine serum (FBS), 2 mM L-glutamine, 100 IU/ml penicillin and 100 µg/ml streptomycin in a humidified incubator with 5% CO_2_ at 37 °C, unless stated otherwise.

Production and purification of PNA-based probes have been described previously in Westerlund *et al*.^13^ and Altai *et al*.^12^. Briefly, the PNA-based probes HP1 and HP2 were synthesized manually using solid phase synthesis with commercially available building blocks. The primary agent, Z_HER2:342_-SR-HP1, was produced by site-specifically attaching HP1 to the anti-HER2 affibody using a sortase A mediated ligation strategy. Z_HER2:342_-SR-HP1 and the secondary agent HP2 were both purified using reversed phase HPLC to a final purity of ≥95% and kept lyophilized at −20 °C until use.

### Labeling

^68^Ge/^68^Ga generator (Cyclotron Co., Obninsk, Russia) was eluted with 0.1 M HCl (prepared from 30% ultrapure HCl from Merck). The generator was eluted with 400 μL fractions of 0.1 M HCl. Fraction 3 containing the maximum radioactivity (ca. 60% of total) was used for labeling.

Lyophilized Z_HER2:342_-SR-HP1 (50 μg, 3.80 nmoles) was dissolved in 50 μL 1.25 M sodium acetate buffer, pH 3.6, then 300 μL of ^68^Ga-containing eluate (165–185 MBq) was added and incubated at 95 °C for 15 min. Then 1000-fold molar excess of Na_4_EDTA (1.44 mg, 3.80 μmoles, 72 μL of 20 mg/mL in 0.625 M sodium acetate, pH 3.6) was added and incubated at 95 °C for 5 min. The radiolabeled compound was purified using NAP-5 size-exclusion column pre-equilibrated with 1% BSA in PBS.

Lyophilized HP2 (50 μg, 9.7 nmoles) was dissolved in 50 μL of 1.25 M sodium acetate buffer, pH 3.6, then 350 μL of ^68^Ga-containing eluate (178–222 MBq) was added and incubated at 95 °C for 15 min. Then 1200-fold molar excess of Na_4_EDTA (4.4 mg, 11.6 μmoles, 110 μL of 40 mg/mL in 0.625 M sodium acetate, pH 3.6) was added and incubated at 95 °C for 5 min. The radiolabeled compound was purified using NAP-5 size-exclusion column pre-equilibrated with 1% BSA in PBS.

Labeling of HP2 with ^177^Lu was performed as described by Altai and coworkers^32^. Briefly, 25 μg (4.9 nmoles) of HP2 was dissolved in 25 μL of 1 M ascorbate buffer, pH 5.5, then 15–20 μL of ^177^LuCl_3_ (7–21 MBq) was added and incubated at 95 °C for 60 min. The radiolabeled compound was purified using NAP-5 size-exclusion column pre-equilibrated with 1% BSA in PBS.

For labeling of affibody DOTA-Z_HER2:2395_ with ^111^In, 30 μg (4.5 nmoles) was dissolved in 30 μL of Milli-Q water, 10 μL of 1.25 M ammonium acetate buffer, pH 4.2, and 100 μL of ^111^InCl_3_ (36–38 MBq) was added and incubated at 60 °C for 30 min. The radiochemical yield of 95 ± 1% was achieved.

Loading of Z_HER2:342_-SR-HP1 with ^nat^Ga and ^nat^In was performed using a 1 to 3 peptide to metal molar ratio. Briefly, to Z_HER2:342_-SR-HP1 (30 μg, 2.3 nmoles) in 30 μL of 1.25 M sodium acetate buffer, pH 3.6, 68 μL of ^nat^GaCl_3_ (1.2 μg, 6.9 nmoles) or 15 μL of ^nat^InCl_3_ (1.5 μg, 6.9 nmoles) in 0.1 M HCl was added and incubated at 95 °C for 15 min for ^nat^Ga labeling and at 90 °C for 30 min for ^nat^In labeling.

The labeling yield and purity were measured by radio-ITLC eluted with 0.2 M citric acid.

### *In vitro* studies

Her2-expressing cell lines used for *in vitro* studies were SKOV3 (1.6 × 10^6^ receptors/cell)^33^, BT474 (1.2 × 10^6^ receptors/cell)^34^ and DU145 (5 × 10^4^ receptors/cell)^35^. Cells were seeded in 3 cm petri dishes (ca. 10^6^ cells/dish), a set of three dishes was used for each data set.

In Her2 binding specificity assay, two sets of dishes were used. A 500-fold excess of non-labeled anti-Her2 Z_HER2:342_ Affibody molecule (1000 nM) was added to the control group of cell dishes to saturate Her2 receptors 5 min before adding the labeled compound. Then ^68^Ga-Z_HER2:342_-SR-HP1 (2 nM) was added to both groups of dishes. The cells were incubated for 1 h in a humidified incubator at 37 °C. Then the medium was collected, the cells were washed and detached by trypsin, the radioactivity in medium and cells was measured to calculate the percent of cell-bound radioactivity.

Pretargeting specificity assay was performed using four sets of cell dishes. To demonstrate the pretargeting, one set of cells was incubated with Z_HER2:342_-SR-HP1 (1 nM) for 1 h at 37 °C and washed. Radiolabeled ^68^Ga-HP2 (10 nM) was added and cells were incubated for 1 h at 37 °C. To show that the pretargeting was Her2-mediated, the second set of cell dishes was incubated with a 500-fold excess of Z_HER2:342_ (1000 nM) for 5 min before the addition of Z_HER2:342_-SR-HP1. Radiolabeled ^68^Ga-HP2 (10 nM) was added and cells were incubated for 1 h at 37 °C. To demonstrate that pretargeting was PNA-mediated, the third set of cell dishes was incubated with Z_HER2:342_-SR-HP1 followed by incubation with a 150-fold excess of non-labeled HP2 (150 nM) for 30 min and then the radiolabeled ^68^Ga-HP2 (10 nM) was added followed by 1 h incubation. In the fourth set the cells were incubated only with ^68^Ga-HP2 (10 nM) to assess non-specific binding. After incubation with ^68^Ga-HP2 the medium was collected, the cells were washed and detached by trypsin to calculate the percent of cell-bound radioactivity.

Cellular retention and processing of ^68^Ga-Z_HER2:342_-SR-HP1 by SKOV3 and DU145 cells was studied during interrupted incubation by an acid-wash method^36^. Cells were incubated with ^68^Ga-Z_HER2:342_-SR-HP1 (1 nM for SKOV3, 0.25 nM for DU145 cells) for 1 h at 4 °C. Then the medium was removed, the cells were washed, new medium was added and the cells were placed in a humidified incubator at 37 °C. At 0.5, 1, 2, 3 and 4 h the medium was collected, cells were washed and treated with 0.2 M glycine buffer containing 4 M urea, pH 2.0, for 5 min on ice. The acidic solution was collected and cells were additionally washed with glycine buffer. The cells were then incubated with 0.5 mL of 1 M NaOH at 37 °C for 10 min and collected with 1 mL of 1 M NaOH. The radioactivity in acidic fractions was considered as membrane-bound, and in the alkaline fractions as internalized.

Cellular retention and processing of Z_HER2:342_-SR-HP1: ^68^Ga-HP2 adduct by SKOV3 and DU145 cells was studied analogously. The cells were incubated with Z_HER2:342_-SR-HP1 (1 nM) for 1 h at 4 °C, then the medium was removed, the cells were washed, ^68^Ga-HP2 (10 nM) was added, and the cells were incubated for 30 min at 4 °C. Then the medium was removed, the cells were washed, new medium was added and the cells were placed in a humidified incubator at 37 °C. At 0.5, 1, 2, 3 and 4 h a group of three dishes was removed from the incubator and treated as described above.

To evaluate the relative binding strength of ^nat^Ga-Z_HER2:342_-SR-HP1 and ^nat^In-Z_HER2:342_-SR-HP1, the half maximal inhibitory concentration (IC_50_) was measured using ^111^In-DOTA-Z_HER2:2395_ in SKOV3 cells. The cells were incubated with ^nat^Ga- or ^nat^In-Z_HER2:342_-SR-HP1 (0–200 nM) in the presence of 1 nM ^111^In-DOTA-Z_HER2:2395_ for 4 h at 4 °C. After incubation the medium was collected, the cells were washed and collected using trypsin to calculate cell-associated activity. The IC_50_ values were determined using GraphPad Prism 7 (GraphPad Software, San Diego, CA, USA).

### Animal studies

Animal studies were planned in agreement with Swedish national legislation concerning protection of laboratory animals and were approved by the Ethics Committee for Animal Research in Uppsala (Permit C 4/2016). All experiments were performed in accordance with the guidelines of the European Community Council Directives 86/609/EEC.

For comparative biodistribution of ^68^Ga-HP2 and ^177^Lu-HP2 a dual-isotope approach was used. Eight female NMRI mice (24 ± 1 g) were intravenously (i.v.) injected with a mixture of ^68^Ga-HP2 and ^177^Lu-HP2 (1 μg in 100 μL of 2% BSA in PBS/mouse, 70 kBq for ^68^Ga-HP2, 45 kBq for ^177^Lu-HP2). At 1 and 2 h mice were anesthesized by an intraperitoneal injection of Ketalar and Rompun solution, followed by euthanasia through cervical dislocation. Blood and organs were collected, weighed and the total radioactivity corresponding to the sum of ^68^Ga and ^177^Lu signals was measured using an open protocol. One day after the first radioactivity measurement (when ^68^Ga has decayed), the ^177^Lu radioactivity was measured in all samples using the same protocol. Subtraction of the ^177^Lu decay-corrected radioactivity from the total signal measured on the first day was considered as ^68^Ga radioactivity. The percent of injected dose per gram of sample (%ID/g) was calculated. Statistical analysis (two-tailed paired t test) was performed using Microsoft Excel 2016 (Microsoft, Redmond, WA, USA).

For tumor implantation, 10^7^ SKOV3 cells or 5 × 10^6^ DU145 cells were subcutaneously injected on the left hind leg of female BALB/c nu/nu mice. The biodistribution experiments were performed two weeks after cell implantation. The average animal weight was 18 ± 1 g in SKOV3 group, 19 ± 1 g in DU145 group. The average tumor weight was 0.08 ± 0.03 g for SKOV3 xenografts, 0.06 ± 0.03 g for DU145 xenografts.

For the biodistribution of the primary agent, mice bearing SKOV3 and DU145 xenografts were injected i.v. with ^68^Ga-Z_HER2:342_-SR-HP1 (100 μg in 100 μL of PBS/mouse, 800 kBq/mouse in SKOV3 group, 1200 kBq/mouse in DU145 group). One hour later, the mice were sacrificed and treated as described above. In the pretargeting approach, mice bearing SKOV3 and DU145 xenografts were injected i.v. with Z_HER2:342_-SR-HP1 (100 μg in 100 μL of PBS/mouse). After 16 h, the mice were injected i.v. with a mixture of ^68^Ga-HP2 and ^177^Lu-HP2 (3.5 μg total in 100 μL of 2% BSA in PBS/mouse, 130 kBq for ^68^Ga-HP2 and 45 kBq for ^177^Lu-HP2). One hour later, the mice were sacrificed and treated as described above. Statistical analysis (two-tailed paired t test) was performed using Microsoft Excel 2016 (Microsoft, Redmond, WA, USA).

### Imaging

Mice bearing SKOV3 and DU145 xenografts were injected i.v. with 100 µg of ^68^Ga-Z_HER2:342_-SR-HP1 (8.2 MBq for SKOV3, 9.1 MBq for DU145). For pretargeting, mice bearing SKOV3 and DU145 xenografts were injected i.v. with 100 µg Z_HER2:342_-SR-HP1 16 h before injection of 3.5 µg of ^68^Ga-HP2 (4.8 MBq for SKOV3, 7.6 MBq for DU145). Immediately before imaging (1 h p.i.), the animals were sacrificed by CO_2_ asphyxiation. PET imaging was performed using Triumph™ Trimodality system (Gamma Medica). The CT scan was performed at the following parameters: field of view (FOV), 8 cm; magnification, 1.48; one frame and 512 projections for 2.13 min. PET data were acquired in list mode during 30 min and reconstructed using OSEM-3D. CT raw files were reconstructed by filter back projection using Nucline 2.03 Software (Mediso Medical Imaging Systems, Hungary). PET and CT dicom files were analyzed using PMOD v 3.12 software (PMOD Technologies, Switzerland).

## Electronic supplementary material

Supplementary information
