# Supplementary material for: Development of an optimal imaging strategy for selection of patients for affibody-based PNA-mediated radionuclide therapy
Source: Sci Rep. 2018 Jun 25;8:9643. doi: 10.1038/s41598-018-27886-0 (PMC6018533; doi:10.1038/s41598-018-27886-0)
Supplement: Supplementary file 1 — Supplementary information [file 41598_2018_27886_MOESM1_ESM.pdf]

Supplementary Information

Development of an optimal imaging strategy for selection of patients for affibody-based PNA-mediated radionuclide therapy

Anzhelika Vorobyeva, Kristina Westerlund, Bogdan Mitran, Mohamed Altai, Sara Rinne, Jens Sörensen, Anna Orlova, Vladimir Tolmachev, Amelie Eriksson Karlström

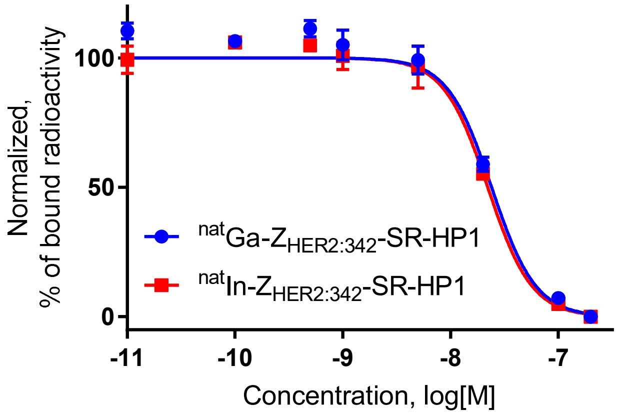

SI Figure 1. Inhibition of ^{111}In-DOTA-Z$_{HER2:2395}$ binding to SKOV3 cells by ^{nat}Ga-Z$_{HER2:342}$-SR-HP1 and ^{nat}In-Z$_{HER2:342}$-SR-HP1. Data is presented as mean ± SD for three cell culture dishes. Error bars might not be seen when they are smaller than symbols.

	1 h		2 h	
	**^{177}Lu-HP2**	**^{68}Ga-HP2**	**^{177}Lu-HP2**	**^{68}Ga-HP2**
**blood**	0.16 ± 0.03	0.21 ± 0.05*	0.03 ± 0.02	0.06 ± 0.01*
**lung**	0.17 ± 0.06	0.28 ± 0.06*	0.11 ± 0.04	0.20 ± 0.08*
**liver**	0.10 ± 0.02	0.23 ± 0.04**	0.07 ± 0.01	0.21 ± 0.02***
**spleen**	0.05 ± 0.03	0.14 ± 0.05	0.01 ± 0.01	0.15 ± 0.09*
**kidney**	4.6 ± 0.5	9 ± 2**	6 ± 2	13 ± 3**
**muscle**	0.07 ± 0.02	0.09 ± 0.03	0.04 ± 0.02	0.06 ± 0.01
**bone**	0.08 ± 0.02	0.11 ± 0.03*	0.04 ± 0.02	0.08 ± 0.04
**GI tract**	0.4 ± 0.1	0.5 ± 0.2	0.3 ± 0.1	0.5 ± 0.1
**carcass**	1.6 ± 0.3	2.3 ± 0.1*	0.8 ± 0.1	1.5 ± 0.3*

SI Table 1. Comparative biodistribution of ^{68}Ga-HP2 and ^{177}Lu-HP2 (3.5 µg total) in female NMRI mice after i.v. injection. The uptake is expressed as % ID/g and presented as an average value from 4 mice ± SD. Data for GI tract with content and carcass are presented as % of ID per whole sample. Symbols show significant difference (* $p < 0.05$, ** $p < 0.01$, *** $p < 0.001$; paired t test) between ^{68}Ga-HP2 and ^{177}Lu-HP2 for each time point.

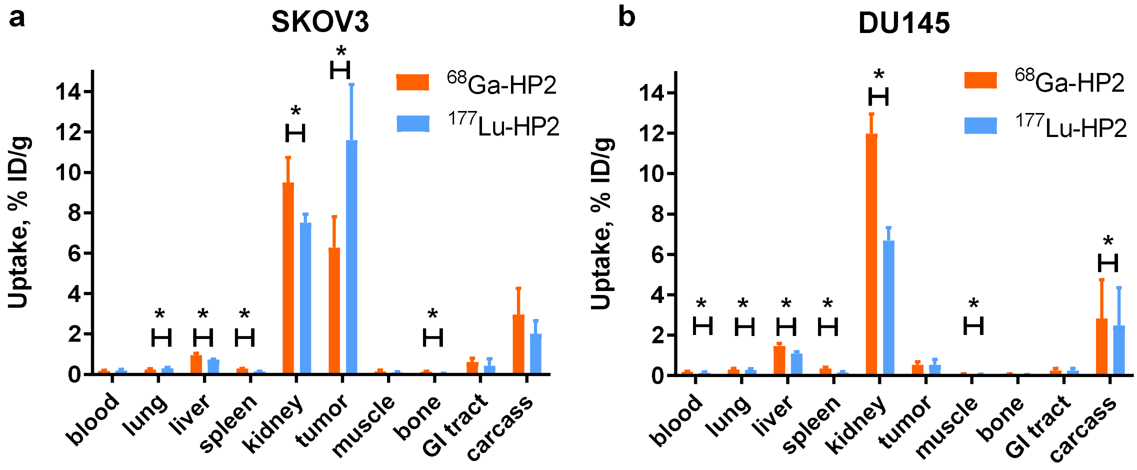

SI Figure 2. Comparison of ^{68}Ga-HP2 and ^{177}Lu-HP2 uptake at 1 h p.i. in BALB/C nu/nu mice bearing SKOV3 (high Her2 expression) (a) and DU145 xenografts (low Her2 expression) (b) pretargeted with $Z_{HER2:342}$-SR-HP1 (100 µg) 16 h before injection of the secondary probes. The uptake is expressed as % ID/g and presented as an average value from 5 mice ± SD. Data for GI tract with content and carcass are presented as % of ID per whole sample. Symbol shows significant difference (* $p < 0.05$; paired t test) between uptake of ^{68}Ga-HP2 and ^{177}Lu-HP2.

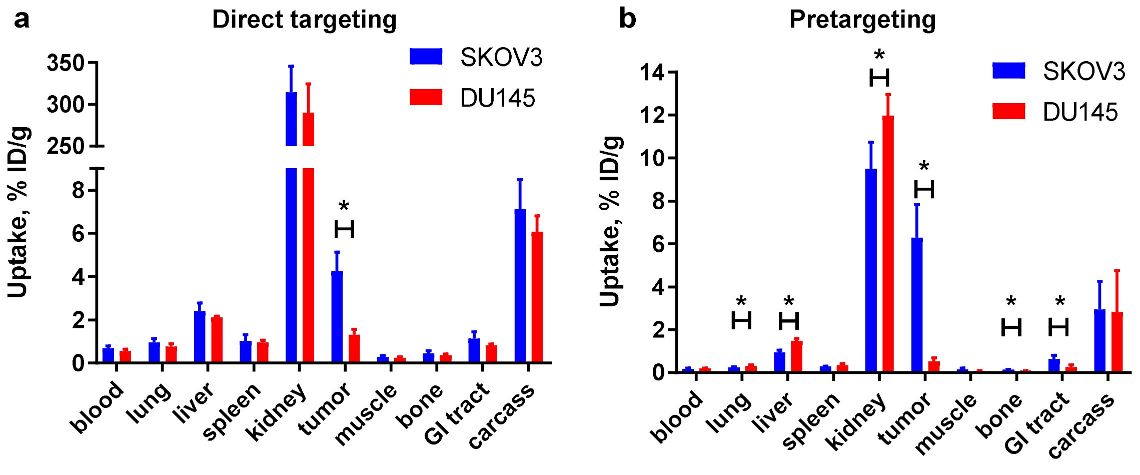

SI Figure 3. Comparison of direct targeting using ^{68}Ga-$Z_{HER2:342}$-SR-HP1 (a) and pretargeting using $Z_{HER2:342}$-SR-HP1 and ^{68}Ga-HP2 (b) in BALB/C nu/nu mice bearing SKOV3 (high Her2 expression) and DU145 xenografts (low Her2 expression) at 1 h p.i. The dose of $Z_{HER2:342}$-SR-HP1 was 100 µg. In pretargeting a mixture of ^{68}Ga-HP2 and ^{177}Lu-HP2 (3.5 µg total) was injected 16 h after the primary agent. The uptake is expressed as % ID/g and presented as an average value from 4 mice ± SD (5 mice ± SD for the pretargeting groups). Data for GI tract with content and carcass are presented as % of injected dose per whole sample. Symbol shows significant difference (* $p < 0.05$; unpaired t test) between uptake in SKOV3 and DU145 groups.
